# Whitefly genomes contain ribotoxin coding genes acquired from plants

**DOI:** 10.1038/s41598-020-72267-1

**Published:** 2020-09-23

**Authors:** Walter J. Lapadula, María L. Mascotti, Maximiliano Juri Ayub

**Affiliations:** Facultad de Química Bioquímica y Farmacia, IMIBIO-SL CONICET, Universidad Nacional de San Luis, Ejército de los Andes 950, D5700HHW San Luis, Argentina

**Keywords:** Evolution, Evolutionary genetics

## Abstract

Ribosome inactivating proteins (RIPs) are RNA *N*-glycosidases that depurinate a specific adenine residue in the conserved sarcin/ricin loop of 28S rRNA. These enzymes are widely distributed among plants and bacteria. Previously, we have described for the first time *RIP* genes in mosquitoes belonging to the Culicidae family. We showed that these genes are derived from a single event of horizontal gene transfer (HGT) from a prokaryotic donor. Mosquito *RIP* genes are evolving under purifying selection, strongly suggesting that these toxins have acquired a functional role. In this work, we show the existence of two RIP encoding genes in the genome of the whitefly *Bemisia tabaci*, a hemiptera species belonging to the Aleyrodidae family distantly related to mosquitoes. Contamination artifacts were ruled out analyzing three independent *B. tabaci* genome databases. In contrast to mosquito *RIP*s, whitefly genes harbor introns and according to transcriptomic evidence are transcribed and spliced. Phylogeny and the taxonomic distribution strongly support that whitefly *RIP* genes are derived from an independent HGT event from a plant source. These results, along with our previous description of RIPs in Diptera, suggest that the acquired genes are functional in these insects and confer some fitness advantage.

## Introduction

Horizontal gene transfer (HGT) is the reproduction-independent transmission of genetic material between organisms of different species. HGT has been reported to have occurred in all the three domains of life and is accepted as an important evolutionary force in prokaryotes^1–3^. On the contrary, its impact on multicellular eukaryotes (e.g. metazoans) is largely controversial^4^. In arthropods, many well-supported HGT events from bacterial or fungal sources have been described^5,6^. Moreover, it has been suggested that HGT played a role in the herbivory of several arthropods and nematodes (see^7,8^ for a review).

Ribosome inactivating proteins (RIPs, EC 3.2.2.22) are RNA *N*-glycosidases depurinating ribosomes in the conserved alpha-sarcin/ricin loop of 28S rRNA, leading to irreversible arresting of protein synthesis^9–11^. RIP encoding genes are widely distributed in plants but scarce within bacterial and fungal lineages^12^. Previously, we demonstrated the presence of *RIP* genes in genomes of mosquitoes belonging to the subfamily Culicinae^13^. This was the first description of the existence of *RIP*s in metazoans. Our research also indicated that these genes are derived from a single HGT event from a bacterial donor species^14^. Moreover, we have shown that, in *Aedes aegypti*, these RIP genes are transcribed and their expression levels are modulated across the developmental stages^15^. Recently, evidence has been accumulated on the role of *Spiroplasma* spp RIPs in the protective mechanisms generated by these endosymbiotic species against arthropod infection by natural enemies (see^16^ for a review).

Here we demonstrate that *RIP* genes are also present in genomes of a second lineage of insects: the hemiptera whiteflies (Aleyrodidae family). Notably, these genes are not closely related to mosquito homologues, instead they form a clade along with plant-derived RIPs. Altogether our results are consistent with two independent HGT events of RIP encoding genes to different insect lineages. Our results suggest that *RIP* genes may fulfill an important functional niche in insects leading to the recurrent selection either from horizontally transferred genetic material or by a symbiotic interaction.

## Results and discussion

### *Bemisia tabaci* genome harbors two RIP encoding genes

**Figure 1:**
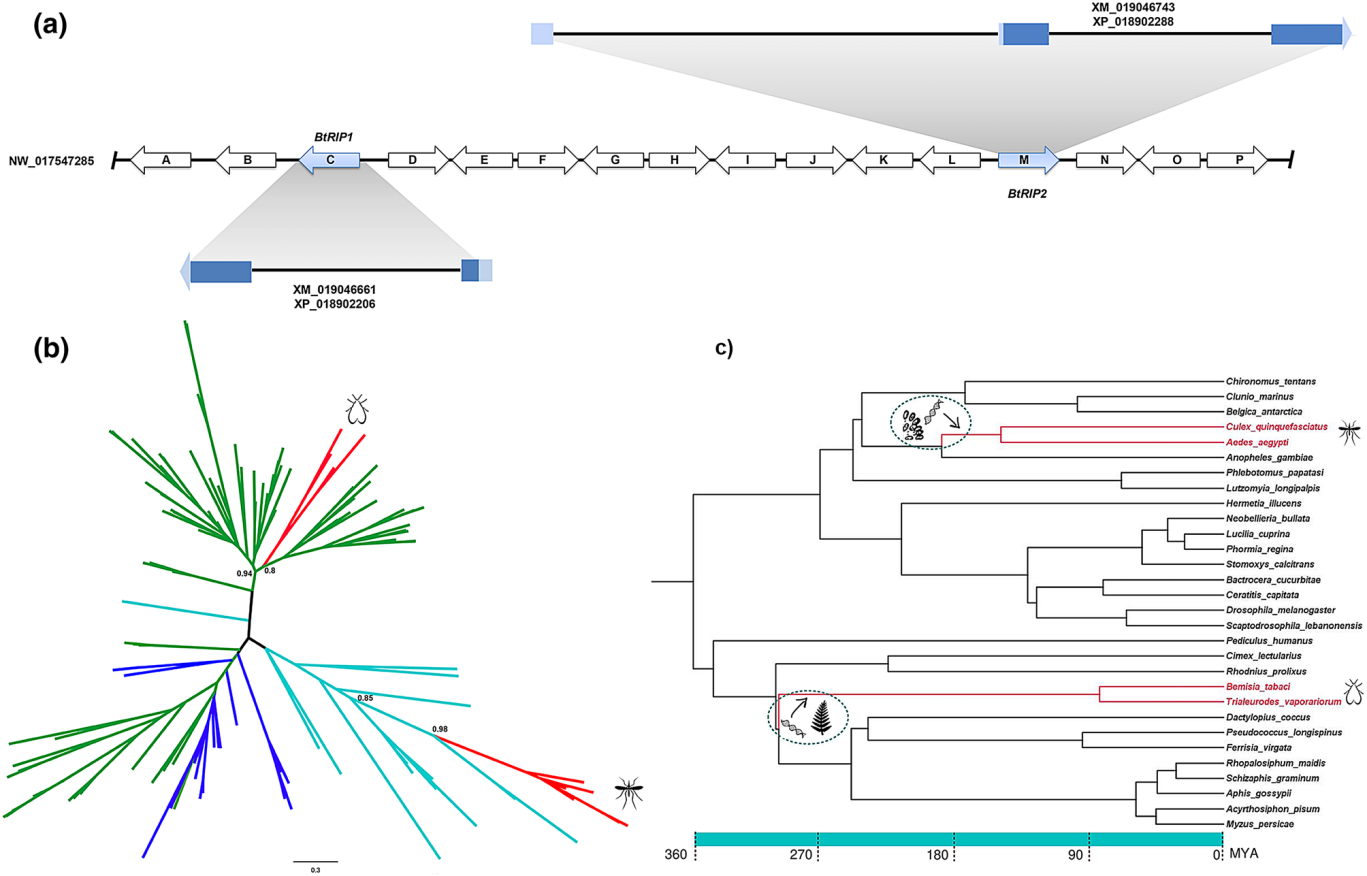
(**A**) Scheme depicting the *B. tabaci* MEAM1 genomic region harboring *BtRIP1* (XM_019046661) and *BtRIP2* (XM_019046743) genes. Arrows depict the genes in the genomic scaffold. The empty arrows show genes surrounding the *BtRIPs* that encode proteins with highest BLAST score to arthropod sequences (see arrow code in Supplementary Table S1). The untranslated (UTRs) and coding regions of mRNA in *BtRIP1* and *BtRIP2* genes are represented with light blue and blue colors, respectively. (**B**) Unrooted phylogeny of *RIP* genes. Branches are colored according to taxonomy: bacteria (light blue), plants (green), fungi (blue), metazoan (red). TBE support values of relevant divergences are shown at nodes. Mosquito and whitefly clades are marked with silhouettes. Fully annotated phylogeny is available as Supplementary Figure S4. (**C**) Phylogeny of selected species from Neoptera orders. The tree including species from Diptera (17), Hemiptera (12) and Psocodea (1) orders with fully sequenced genomes was constructed with the TimeTree knowledge-base^33^. Insects harboring *RIP* genes are shown in red branches. The two independent HGT events are graphically represented at the estimated time windows, with the hypothetical donors shown as silhouettes. Time in million years ago (MYA) is indicated at the bottom.

In the course of routine database searches, we found two RIP encoding sequences in the hemiptera *B. tabaci* MEAM 1 (henceforth named *BtRIP1* and *BtRIP2*). Figure 1A shows a schematic representation of the genomic scaffold (NW_017547285) harboring these genes. In contrast to *RIP* genes from mosquitoes, *BtRIP1* and *BtRIP2* contain one and two introns, respectively. This excludes the chance of bacterial contamination, a possible artifact of genome sequencing projects^17,18^. BLAST analyses of all encoding protein sequences surrounding the *RIP* genes yielded maximum scores with arthropod annotated proteins (Supplementary Table S1). In contrast, BtRIPs showed maximum sequence identity to plant RIPs, and marginal identity to their mosquito homologues (around 24%).

We further confirmed the existence of these *Bemisia* newly discovered genes by analyzing two additional, independent genome contigs belonging to the *B. tabaci* SSA-ECA^19^ (PGTP01000858) and *B. tabaci* MED/Q^20^ (ML134445) assemblies. Supplementary Figure S1 shows synteny analysis of the genomic regions including *RIP* genes in these three subspecies of *B. tabaci* (with the exception of *BtRIP1* in the *Bemisia tabaci* SSA1 due to a 3 kb gap in the corresponding scaffold), ruling out possible artifacts arising from sequence assembly. Supplementary Table S1 summarizes this information.

*BtRIP1* gene is 3,466 bp and includes two exons of 273 bp and 517 bp and a single intron of 2,730 bp. The mRNA (790 bp) shows 5′ and 3′UTRs of 92 bp and 86 bp, respectively. The mature mRNA encodes a protein of 185 amino acids (Fig. 1A). The *BtRIP2* gene extends over 5,974 bp: three exons (156 bp, 354 bp and 563 bp) and two introns (3,156 bp and 1,745 bp). The 5´ UTR (186 bp) is formed by the whole first exon and 30 bp of the second exon (Fig. 1A). The 3´UTR has 65 bp of the last exon. The mature mRNA encodes a protein of 273 amino acids. Sequence alignment and structural modeling of *B. tabaci* predicted proteins revealed that all the characterized residues forming the active site are conserved. Interestingly, BtRIP1 is remarkably smaller than average RIPs. This is caused by a *N*-terminal shortening. The functional relevance of this shortening is not clear from the structural model since although a big open cavity is generated, the active site architecture is conserved. Other shorter *N*-terminal deletions are observed in functional RIPs as lychinin (Protein Data Bank; PDB 2G5X). BtRIP2 is predicted to conserve the architecture and all secondary structure elements canonical of functional RIPs such as momordin (PDB 5CF9) or trichosantin (PDB 1QD2) (Supplementary Figure S2).

By using transcriptomic data (kindly provided by Dr. Zhangjun Fei^21^), we analyzed the expression level of *BtRIPs* in comparison with the full set of genes. Transcripts for both genes were found. In particular, *BtRIP2* expression level is in the top quartile of *B. tabaci* genes, suggesting a relevant functional role for this protein (Supplementary Figure S3).

### *Trialeurodes vaporariorum* draft genome harbors three non-annotated RIP encoding sequences

In December 2019, the draft genome sequence of *T. vaporariorum*, a whitefly closely related to *B. tabaci* was released. We downloaded the genome data and performed tBLASTn searches using *B. tabaci* RIPs as queries. We found three contiguous RIP encoding sequences in the same scaffold (VJOP01000134); namely *TvRIP1* (nt 308,541–309,308), *TvRIP2* (nt 315,008–315,853) and *TvRIP3* (nt 327,149–327,907). The amino acid identity among TvRIPs ranged from 38 to 72%. These proteins showed 30–35% identity when compared to BtRIPs.

### Whitefly *RIP* genes are phylogenetically closer to plant than to mosquito homologues

Next, we performed phylogenetic inferences using the recently discovered whitefly RIPs along with a representative dataset of RIP sequences from our previous works^13,14^. The phylogenetic relationships among whitefly RIPs and previously characterized homologues are expected to shed light on the evolutionary origin and history of these genes and their possible relationship with homologous genes from dipterans. Notably, as can be seen in Fig. 1B, whitefly RIPs are embedded into a clade of plant sequences (Transfer Boostrap Expectation; TBE = 0.94). These RIPs are distantly related to mosquito RIPs, which on the contrary are gathered in a clade of bacterial homologues (TBE = 0.85). *B. tabaci* and *T. vaporariorum* genes form sister clades, revealing that the duplication events occurred after divergence of these species yielding two and three different paralogs, respectively (Supplementary Figure S4).

### Whitefly RIPs are derived from a plant genome via a single HGT event

To determine the presence/absence of *RIP*s in other insects, we performed homology searches using whitefly RIPs as queries in complete genomes of insects other than *B. tabaci* and *T. vaporariorum* (797 assemblies with full representation). No hits were retrieved even in other hemiptera species. This fact, along with the phylogenetic inferences, indicates that these genes are not derived from vertical inheritance through the insect lineage. Instead, they originate from an independent HGT event of a plant *RIP* gene to an insect genome. Figure 1C shows the phylogenetic relationships among selected Neoptera species. As it can be observed only two clades harbor *RIP* genes: the Diptera lineage including Culicini and Aedini tribes and, the Hemiptera lineage that includes *B. tabaci* and *T. vaporariorum* species.

As previously reported, Diptera *RIP* genes are derived from a single HGT event which took place between the divergence of *Anopheles* and *Culex/Aedes* lineages (around 190 MYA) and before the separation of *Aedes* and *Culex* genus (150 MYA) (Fig. 1C). In a similar way, whitefly *RIP* genes seem to have been also acquired by HGT but from an eukaryotic, plant genome source. The absence of *RIP* genes in other hemiptera species suggests that HGT took place in the lineage of suborder Sternorrhyncha before the divergence between *B. tabaci* and *T. vaporariorum species* (in the range of 300–80 MYA).

We have previously postulated that HGT from bacteria to mosquitoes could have been facilitated by the weakness of the Weisman barrier (i.e. the physical separation between somatic and germinal cells in multicellular organisms) at early developmental stages. Feeding mosquito larva on bacterial species in their ecological niches is consistent with the acquisition of a *RIP* gene from prokaryotes^14^. The findings presented in this work seem to support this hypothesis, since early development stages whiteflies feed on plant sap. Therefore, our previous and current findings pinpoint that in addition to phylogenetic inconsistencies and taxonomic distribution, developmental and ecological features of animal species should be carefully analyzed when investigating and testing the plausibility of HGT events.

### Whitefly *RIP* genes have evolved under purifying selection

After the integration in the genome of ancestral whitefly, *RIP* genes have not been lost by genetic drift over 80 million years of evolution. This strongly suggests that these genes are playing a functional role in their hosts. We tested this hypothesis by analyzing the nonsynonymous/synonymous substitution rates ω(dN/dS). As expected, this analysis yielded a global ω value of 0.34 consistent with evolution under purifying selection.

We have previously shown that almost all sequenced metazoan genomes are devoid of *RIP* genes^13,14^, the only two exceptions are insect lineages of Culicidae^14^ and Aleyrodidae (reported in this work) families. Both of these lineages have incorporated RIP encoding genes via HGT. These genes are actively transcribed and evolve under purifying selection. Based on this evidence we propose that after integration in their host genomes, *RIP* genes have a positive impact on species fitness. The defensive role of *Spiroplasma* encoded RIPs reported in *Drosophila*^16^ suggests that exogenous insect RIPs might play a similar protective role.

## Methods

### Homology searches and sequence analyses

BLASTp and tBLASTn homology searches were performed under default parameters on metazoan databases, using as queries a previously reported set of RIP sequences^14^. Two automatically annotated protein sequences (GenBank: XP_018902206, XP_018902288) were retrieved from the *B. tabaci* MEAM 1 genome database. Pfam analysis was performed to confirm the presence of RIP domain (PF00161)^22^. Homology models were generated using Swiss-Model server^23^ and visualized in PyMOL. Active site residues^24^ were detected by sequence alignment to momordin (PDB 3MY6). The *T. vaporariorum* non-annotated draft genome (GenBank: VJOP01000000) was downloaded and standalone tBLASTn searches performed using *B. tabaci* RIPs (GenBank: XP_018902206, XP_018902288) as queries.

### Synteny analysis

The contig region containing *RIP* genes from *B. tabaci* MEAM 1 (NW017547285)^21^ was compared employing Mauve software^25^ (version 2.4.0, available at https://darlinglab.org/mauve) to the corresponding genomic fragments of other members of the *B. tabaci* complex; namely *B. tabaci* SSA-ECA (PGTP01000858)^19^ and *B. tabaci* MED/Q (ML134445)^20^.

### Transcriptomic data analysis

The full RNA-seq dataset expressed as Reads Per Kilobase of transcript per Million mapped reads (RPKM) of *B. tabaci* MEAM 1 was kindly provided by Dr. Zhangjun Fei^21^. The logarithm of the average level (among different experimental conditions) of gene expression for *BtRIP1* (Gene ID: Bta13094) and *BtRIP2* (Gene ID: Bta13103) were plotted along with box and whisker graph showing quartiles for expression level of the whole set of *B. tabaci* genes using GraphPad Prism version 5.00 for Windows.

### Multiple sequence alignment and phylogenetic inferences

The new *B. tabaci* and *T. vaporariorum* RIP protein sequences were incorporated to our previous dataset^13^. The conserved region in the RIP domain (from Y14 to S196 residues according to trichosanthin (GenBank: AAT91090) was selected for alignment as previously reported^13,14,26^. Multiple sequence alignment (MSA) was constructed using MAFFT 7 server^27^ employing BLOSUM 30 as scoring matrix. Poorly aligned regions were trimmed as blocks. This MSA containing 125 sequences and 199 sites was used to perform phylogenetic analysis by Maximum Likelihood in RAxML (version 8.2.10, available at https://github.com/stamatak/standard-RAxML)^28^. The WAG substitution matrix was selected using ProtTest 3.4^29^. To estimate the robustness of the phylogenetic inference 500 rapid bootstrap (BS) were selected. Transfer bootstrap expectation was calculated in BOOSTER^30^. Phylogenetic relationships and divergence times among species were obtained from TimeTree knowledge-base^31^. FigTree (version 1.4.2, available at https://tree.bio.ed.ac.uk/software/figtree) was used to visualize and edit the trees.

### Selection pressure analysis

The omega (ω) ratio between nonsynonymous/synonymous substitution rates was calculated with codeml in PAML 4.9^32^. This analysis was performed under the one ratio model (M0) with κ = 2.096, α = 5.234 under 4 gamma categories (parameters calculated in jModelTest 2.1.10). The codon alignment used as input was created in PAL2NAL and the protein based tree in MAFFT by (Neighbor Joining) NJ method.

## Supplementary information

Supplementary Information
