# Supplementary material for: Whitefly genomes contain ribotoxin coding genes acquired from plants
Source: Sci Rep. 2020 Sep 23;10:15503. doi: 10.1038/s41598-020-72267-1 (PMC7511414; doi:10.1038/s41598-020-72267-1)
Supplement: Supplementary file 1 — Supplementary Information [file 41598_2020_72267_MOESM1_ESM.pdf]

Whitefly genomes contain ribotoxin coding genes acquired from plants

Walter J. Lapadula*, María Laura Mascotti, Maximiliano Juri Ayub*

IMIBIO-SL CONICET, Facultad de Química Bioquímica y Farmacia, Universidad Nacional de San Luis, Ejército de los Andes 950, D5700HHW, San Luis, Argentina.

*Correspondence and requests for materials should be addressed to W.J.L (e-mail: wlapadula@gmail.com) or M.J.A. (e-mail: mjuriayub@hotmail.com)

Supplementary information

Supplementary Table 1. Contig region containing BtRIP genes. Annotated proteins GenPept cdes of *B. tabaci* are shown along with their location in different genome databases. The highest identity hit in BLASTP searches for each protein is indicated, along with the identity percent and coverage percent.

							NW_017547285			PGTP01000858			ML134445		
							Bemisia tabaci MEAM1			*Bemisia tabaci* SSA1			*Bemisia tabaci* MED-Q		
Annotation	Arrow code Figure 1 A	GenPept	Closest BLASTP	Organism	Id (%)/QC (%)	Orientation according to NW_017547285	Initiation	End	Gen length (bp)	Initiation	End	Gen length (bp)	Initiation	End	Gen length (bp)
elongation of very long chain fatty acids protein 7-l	A	XP 018902197	VVC28064	Cinara cedri	48/93	-	985766	992173	6407	199829	206692	6863	165303	172259	6956
elongation of very long chain fatty acids protein 7-l	B	XP_018902200	XP_015364617	Diuraphis noxia	51/97.7	-	993246	1009513	16267	186383	198288	11905	179925	187284	7359
ribosome-inactivating protein	C	XP 018902206	XP 002533608	Ricinus communis	33/58.9	-	1017949	1021233	3284	NA	NA	NA	149674	152264	2590
rabenosyn-5	D	XP 018902267	XP 014271881	Halyomorpha halys	50/94.4	+	1034969	1044873	9904	152706	162699	9993	123566	133452	9886
protein YIPF6	E	XP_018902269	XP_023722963	Cryptotermes secundus	67/98.3	-	1046825	1051805	4980	146162	151093	4931	116371	121279	4908
TFIID subunit 1-like	F	XP 018902266	PNF40462	Cryptotermes secundus	66/99.7	+	1052478	1080060	27582	118682	145465	26783	89677	115706	26029
HMGIC fusion partner-like 3	G	XP 018902290	XP 023715865	Cryptotermes secundus	71/100	-	1087672	1089807	2135	109245	111375	2130	79739	81871	2132
ubiquitin carboxyl-terminal hydrolase 16	H	XP_018902226	XP_030036242	Manduca sexta	34/100	+	1093723	1108974	15251	88677	105921	17244	60327	76392	16065
coiled-coil domain-containing protein 93 isoform X1	I	XP_018902228	XP_022184911	Nilaparvata lugens	47/98.6	-	1113316	1128262	14946	69713	83924	14211	40005	54622	14617
:hoxy-6-polyprenyl-1,4-benzoquinol methylase, mitochon	J	XP_018902233	RZF39836	Laodelphax striatellus	70/92.1	+	1128922	1129800	878	68196	69074	878	38481	39359	878
2-phosphoxylose phosphatase 1	K	XP_018902230	XP_023717146	Cryptotermes secundus	45/98.6	-	1133122	1136731	3609	61248	64880	3632	31861	36346	4485
TBC1 domain family member 13	L	XP_018902284	XP_023712160	Cryptotermes secundus	72/96.9	-	1143805	1152910	9105	46521	53455	6934	20863	NA	NA
ribosome-inactivating protein luffaculin 1-like	M	XP 018902288	AAS15568	Gynostemma pentaphyllum	34/64.4	+	1158736	1161290	2554	37269	40465	3196	10307	12611	2304
LIM/homeobox protein Awh isoform X1	N	XP 018902192	RZF48072	Laodelphax striatellus	78/93.9	+	1230384	1340031	109647	NA	NA	NA	NA	NA	NA
ubiquitin carboxyl-terminal hydrolase isozyme L3	O	XP_018902296	XP_023724885	Cryptotermes secundus	98.7	-	1349895	1358235	8340	NA	NA	NA	NA	NA	NA
uncharacterized protein CG1161 isoform X1	P	XP 018902286	XP 024214830	Halyomorpha halys	60/97.5	+	1358825	1363863	5038	NA	NA	NA	NA	NA	NA

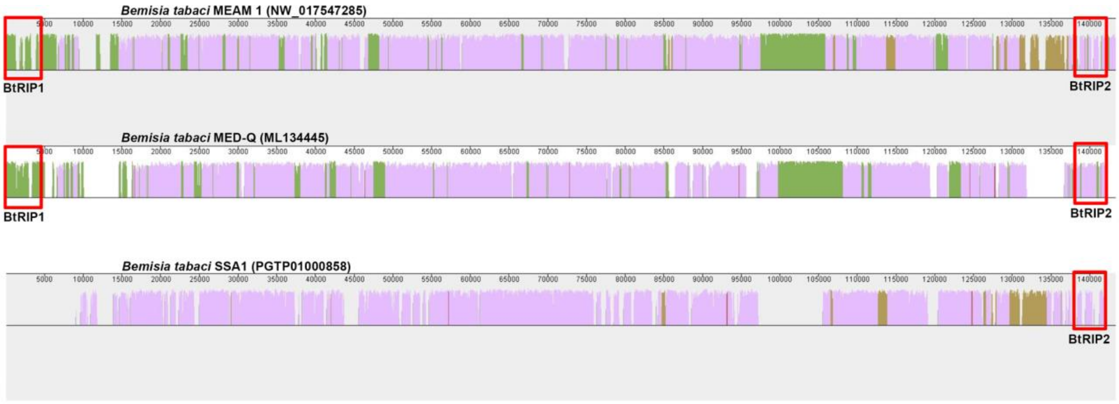

Figure S1. Scheme depicting the *B. tabaci* genomic region harboring *BtRIP1* and *BtRIP2* genes. The contig region containing *RIP* genes from *B. tabaci* MEAM 1 (NW017547285), *B. tabaci* MED/Q (ML134445) and *B. tabaci* SSA-ECA (PGTP01000858) were compared using MAUVE software [1]. Sections of the similarity plot colored in violet are conserved among all three genomes; portions in green are conserved among the MEAM 1 and MED-Q sequences, segments conserved among MEAM 1 and SSA 1 are colored in brown. Empty spaces represent regions with no sequence available. Regions corresponding to *BtRIP1* and *BtRIP2* are indicated with red boxes.

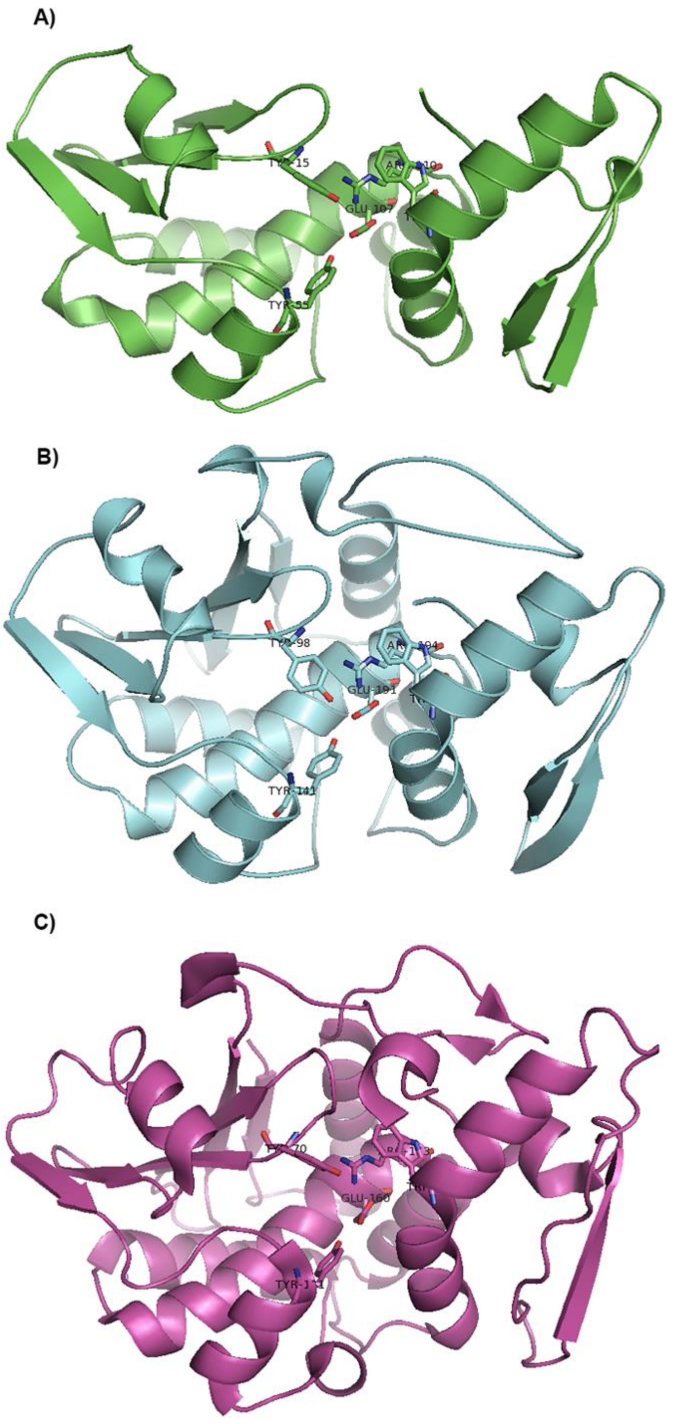

Figure S2. Homology models of BtRIP1 and BtRIP2. Models were constructed in Swiss-Model server [2]. Structures of momordin (PDB 3MY6 or 4YP2) displaying 30-32% sequence identity were used as templates for both BtRIP1 and BtRIP2. **A.** Homology model of BtRIP1 (green). **B.** Homology model of BtRIP2 (cyan) **C.** Structure of momordin PDB 3MY6 (magenta). Active site residues (Y, Y, E, R, W) are numbered and shown as sticks.

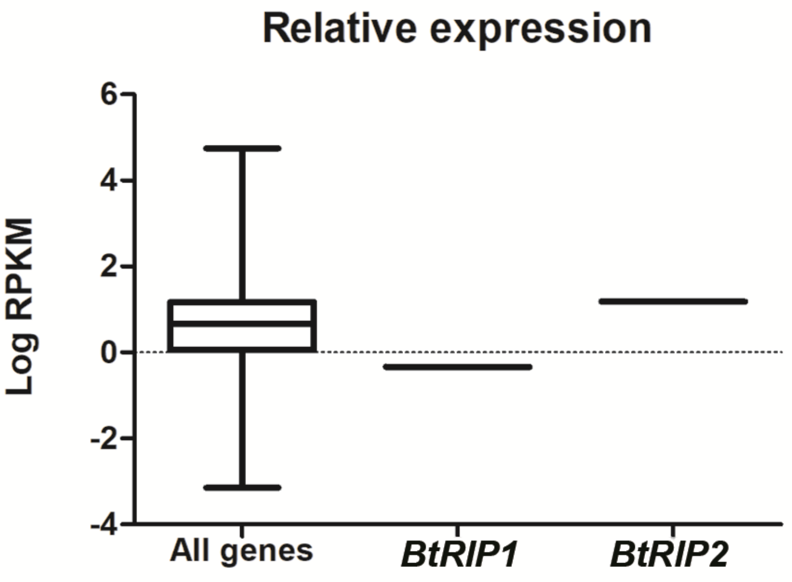

Figure S3. Expression level of *BtRIPs*. Data provided by Dr. Dr. Zhangjun Fei were plotted as log of RPKM. Values of *BtRIP1* and *BtRIP2* were compared with the whole set of *B. tabaci* genes according to Chen *et al*. [3]. The box-and-whisker plot shows expression levels as quartiles.

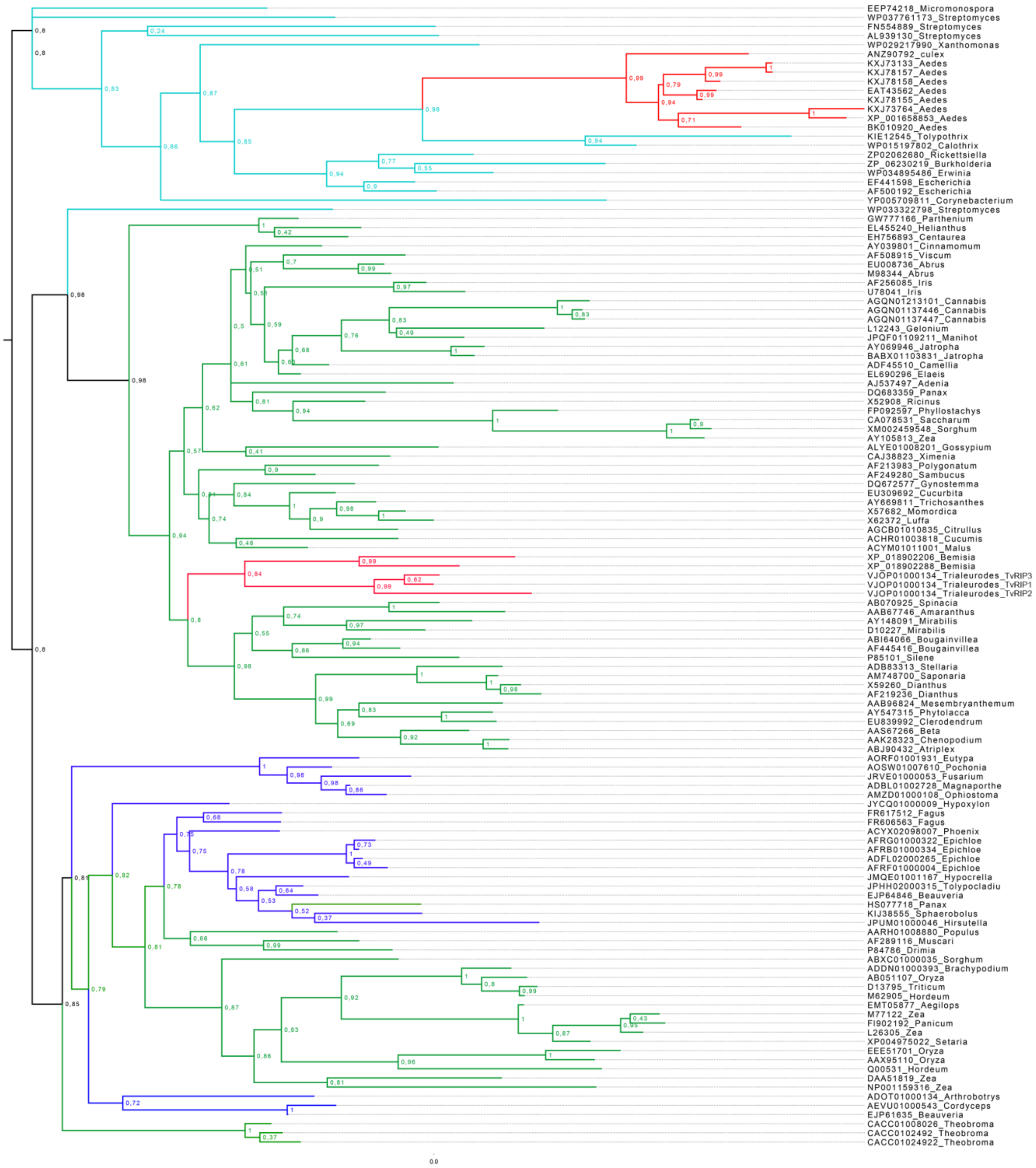

Figure S4. RIPs fully annotated phylogeny. Midpoint rooted phylogeny of RIP sequences. Tree was constructed in RAXML 8.2.12. Branches are colored according to taxonomy: bacteria (cyan), green (plants), fungi (blue), metazoan (red). 500 rapid bootstrapping were performed and then transformed to TBE in BOOSTER. TBE support values are shown in nodes. Genus and GenBank accession codes are given for each taxa.

References

1. Darling, A.C., et al., *Mauve: multiple alignment of conserved genomic sequence with rearrangements.* Genome Res, 2004. **14**(7): p. 1394-403.
2. Waterhouse, A., et al., *SWISS-MODEL: homology modelling of protein structures and complexes.* Nucleic Acids Res, 2018. **46**(W1): p. W296-W303.
3. Chen, W., et al., *The draft genome of whitefly Bemisia tabaci MEAM1, a global crop pest, provides novel insights into virus transmission, host adaptation, and insecticide resistance.* BMC Biol, 2016. **14**(1): p. 110.
